# The effect of TNF-α inhibitor treatment on microRNAs and endothelial function in collagen induced arthritis

**DOI:** 10.1371/journal.pone.0264558

**Published:** 2022-02-25

**Authors:** Sulè Gunter, Frederic S. Michel, Serena S. Fourie, Mikayra Singh, Regina le Roux, Ashmeetha Manilall, Lebogang P. Mokotedi, Aletta M. E. Millen

**Affiliations:** School of Physiology, Faculty of Health Sciences, University of the Witwatersrand, Johannesburg, South Africa; University of Calabria, ITALY

## Abstract

Chronic inflammation causes dysregulated expression of microRNAs. Aberrant microRNA expression is associated with endothelial dysfunction. In this study we determined whether TNF-α inhibition impacted the expression of miRNA-146a-5p and miRNA-155-5p, and whether changes in the expression of these miRNAs were related to inflammation-induced changes in endothelial function in collagen-induced arthritis (CIA). Sixty-four Sprague-Dawley rats were divided into control (n = 24), CIA (n = 24) and CIA+etanercept (n = 16) groups. CIA and CIA+etanercept groups were immunized with bovine type-II collagen, emulsified in incomplete Freund’s adjuvant. Upon signs of arthritis, the CIA+etanercept group received 10mg/kg of etanercept intraperitoneally, every three days. After six weeks of treatment, mesenteric artery vascular reactivity was assessed using wire-myography. Serum concentrations of TNF-α, C-reactive protein, interleukin-6, vascular adhesion molecule-1 (VCAM-1) and pentraxin-3 (PTX-3) were measured by ELISA. Relative expression of circulating miRNA-146a-5p and miRNA-155-5p were determined using RT-qPCR. Compared to controls, circulating miRNA-155-5p, VCAM-1 and PTX-3 concentrations were increased, and vessel relaxation was impaired in the CIA (all p<0.05), but not in the CIA+etanercept (all p<0.05) groups. The CIA group had greater miRNA-146a-5p expression compared to the CIA+etanercept group (p = 0.005). Independent of blood pressure, miRNA-146a-5p expression was associated with increased PTX-3 concentrations (p = 0.03), while miRNA-155-5p expression was associated with impaired vessel relaxation (p = 0.01). In conclusion, blocking circulating TNF-α impacted systemic inflammation-induced increased expression of miRNA-146a-5p and miRNA-155-5p, which were associated with endothelial inflammation and impaired endothelial dependent vasorelaxation, respectively.

## Introduction

Rheumatoid arthritis (RA) is associated with a greater risk of cardiovascular events and mortality [1]. Subclinical vascular changes including endothelial dysfunction, arteriosclerosis and atherosclerosis are more prevalent in RA compared to the general population and contribute to the increased cardiovascular disease (CVD) prevalence in RA [2]. Systemic inflammation is associated with impaired vascular function, independent of traditional risk factors in RA [3]. The mechanisms whereby systemic inflammation impairs vascular function are currently under investigation [4].

One of the earliest subclinical cardiovascular pathophysiological features observed in states of high-grade inflammation is endothelial activation [5]. Indeed, inflammation-induced endothelial activation and dysfunction are considered important early predictors of atherosclerotic CVD, arterial stiffness and heart failure [6–8]. Chronic systemic inflammation in RA reportedly modifies the phenotype of endothelial cells, leading to functional and structural alterations in both endothelial and smooth muscle layers [4]. Increased circulating cytokine concentrations promote endothelial activation through increased expression of vascular adhesion molecules, including vascular adhesion molecule-1 (VCAM-1) [9]. VCAM-1 enables leukocyte binding and migration to the arterial wall intima, making it a sensitive marker of endothelial activation and early atherosclerosis [10, 11]. Circulating cytokines additionally induce a concomitant accumulation of reactive oxygen species that drives endothelial inflammation [12]. Recently, pentraxin-3 (PTX-3), an acute phase protein that is produced under inflammatory conditions by various stromal and myeloid cells, has been identified as highly sensitive marker of local vessel inflammation [13]. Local production of PTX-3 impairs endothelial function and amplifies vascular inflammation [14]. Furthermore, endothelial inflammation reduces nitric oxide (NO) bioavailability which results in impaired endothelial-dependent vasodilation and ultimately endothelial dysfunction [15].

Chronic systemic inflammation alters the signaling pathways that govern gene expression of endothelial function markers [16]. In this regard, microRNAs (miRNAs) regulate gene expression by inhibiting the translation of messenger RNA or inducing mRNA degradation [16]. Increasing evidence suggests that high-grade inflammatory states, such as RA, may cause inappropriate expression of miRNAs [17, 18]. MiRNA-155-5p and miRNA-146a-5p expression is strongly induced through TNF-α mediated signalling pathways [18, 19]. MiRNA-155-5p and miRNA-146a-5p expression is dysregulated in RA and have been implicated in the disease pathogenesis [20] and disease activity [21]. In non-RA populations, miRNA-155-5p is involved in endothelium-dependent vasorelaxation, altered endothelial permeability and atherosclerotic progression [22, 23], while miRNA-146a-5p promotes inflammation-induced senescence in vascular remodelling cells [24]. The role of these miRNAs in the development of inflammation-induced endothelial dysfunction in RA is uncertain. Dysregulated expression of miRNA-146a-5p and miRNA-155-5p may present an alternative mechanism whereby vascular integrity is compromised [17], and hence may serve as promising therapeutic targets in RA [25].

TNF-α inhibition therapy successfully slows disease progression and prevents joint damage in the management of RA [26]. However, its effectiveness in simultaneously controlling CVD risk is controversial [27]. The effect of TNF-α inhibitor treatment on endothelial function in RA patients are inconsistent [28, 29]. The confounding effects of combination therapy and other cardiovascular drugs limit our understanding of the effects of TNF-α inhibitor treatment on endothelial function in RA [29]. Moreover, miRNA expression may be induced through several cytokines and the effects of TNF-α inhibition on the expression of miRNAs associated with endothelial dysfunction is uncertain. Therefore, the independent effects of TNF-α inhibition on the regulation of endothelial function in high-grade inflammation requires elucidation. The present study aimed to determine whether TNF-α monotherapy impacts the expression of miRNA-146a-5p and miRNA-155-5p, and whether altered expression of these miRNAs is associated with endothelial dysfunction and impaired vascular reactivity in collagen-induced arthritis (CIA).

## Materials and methods

### Study design and animal treatment

All experimental procedures were approved by the Animal Ethics Screening Committee (AESC) of the University of the Witwatersrand (AESC number: 2017/03/21C and 2019/02/10C) and complied with the Guide for the Care and Use of Laboratory Animals. Three-month-old, Sprague-Dawley rats were housed individually in cages in temperature-controlled rooms (23 ± 2°C), with a 12-hour light-dark cycle and allowed free access to food and water. Rats were habituated to the housing conditions for two weeks, where blood pressure and body weight were measured, as previously described [30]. Following the two-week habituation period, rats were randomly divided into the control (n = 24, 9 females and 15 males), CIA (n = 24, 12 females and 12 males) and CIA+etanercept (n = 16, 8 females and 8 males) groups. Systemic inflammation was induced in the CIA and CIA+etanercept groups using bovine type II collagen dissolved in incomplete Freund’s adjuvant, as previously reported [30]. To ensure a high incidence of arthritis, booster injections (0.1ml) were given after seven and 21 days. All collagen-treated rats exhibited paw inflammation as previously described [30]. Upon the first signs of inflammation, approximately 3–4 weeks after CIA was first induced, rats in the CIA+etanercept group received intraperitoneal injections of etanercept (Enbrel), a TNF-α inhibitor (Roche, Basel, Switzerland) at a dosage of 10 mg/kg every third day for six weeks [30]. Following the onset of arthritis, rats in the CIA and CIA+etanercept groups received subcutaneous injections of 1-4mg/kg Tramadol (Zydus Healthcare, Johannesburg, South Africa), for pain management. The present study included 64 rats from a larger study [30], wherein miRNA expression and markers of endothelial and vascular function were measured.

### Vascular reactivity

Following six-weeks of drug treatment, rats were anaesthetised using ketamine and xylazine at a dosage of 100mg.kg^-1^ and 5mg.kg^-1^, respectively. Rats were terminated by thoracotomy. Vascular reactivity was measured in the mesenteric artery, as previously described [31]. Briefly, the mesentery was removed and cleaned. Second branches of the mesenteric artery were cut into 2 mm rings and suspended in a wire myograph (model 610M; Danish Myo Technology, Aahrus, Denmark). Artery rings were exposed to potassium chloride (KCl, 80 mM) to establish the maximal reference contraction. Thereafter, preparations were exposed to increasing concentration of KCl and phenylephrine (Phe) to determine the contraction responses as a percentage of the maximal contraction. Preparations were then exposed to increasing concentrations of acetylcholine (Ach), an endothelium-dependent vasodilator and sodium nitroprusside (SNP), an endothelium-independent vasodilator during phenylephrine (Phe, 10 μM) induced contractions. Vascular relaxation was expressed as a percentage of the baseline tension during contractions induced by Phe (10 μM).

### Markers of circulating and endothelial inflammation

After thoracotomy, blood was collected in blood collection tubes and allowed to clot for two hours. Thereafter, samples were centrifuged, serum was collected and stored in cryotubes at -80°C until assayed. Individual samples were visually examined for hemolysis by comparing the appearance of the serum with a specimen integrity chart based on colour [32]. Commercially available enzyme-linked immunosorbent assay (ELISA) kits (Elabscience Biotechnology Co. Ltd, Wuhan, China) were used to measure serum levels of tumor necrosis factor-alpha (TNF-α), interleukin 6 (IL-6) and C-reactive protein (CRP). In a subgroup of animals (n = 40) serum levels of vascular adhesion molecule-1 (VCAM-1) and pentraxin-3 (PTX-3) were measured using ELISA. The lower detection limit for TNF-α, IL-6, CRP, VCAM-1 and PTX-3 were 78 pg/ml, 62 pg/ml, 0.3 ng/ml, 12.5 pg/ml and 0.16 ng/ml, respectively. The interassay and intra-assay coefficients of variation were below 10% for all kits.

### MicroRNA expression

Total RNA was extracted from serum using the MagMAX™ mirVana™ Total RNA Isolation Kit (Thermo Fisher Scientific, Waltham, MA). cDNA templates were prepared using TaqMan™ Advanced miRNA cDNA synthesis kits (Thermo Fisher Scientific, Waltham, MA). Production of cDNA (>1000ng/μl) was confirmed using a NanoDrop OneC spectrophotometer (Thermo Fisher Scientific, Waltham, USA).

Real-time quantitative PCR (qPCR) was performed using cDNA (~1μg), pre-designed VIC labelled probe mixes for the reference miRNA (miRNA-191-5p, 0.25 μL, Taqman assay ID: rno480971_mir), the miRNA of interest (0.5 μL Taqman Advanced miRNA Assay), Taqman Fast Advanced Master Mix (5 μL) and 3.25 μL of RNase-free water, to make up a total volume of 10μL per reaction well.

Comparative miRNA expression was assessed in duplicate for miR146a-5p (Taqman assay ID: rno481451) and miRNA-155-5p (Taqman Assay ID: rno480953_mir) in duplex reactions with the endogenous control. Although SNU6 and RNU6 are conventionally used as endogenous controls, recent reports showed that the expression of these genes are unstable [33, 34]. SnU6 and RNU6 do not reflect the biochemical character of miRNA molecules in terms of their transcription, processing and tissue specific expression patterns and may respond differently to qPCR techniques [35, 36]. Based on comparison to another potential miRNA reference gene (miR26a-5p), miR191-5p was found to be most consistently expressed in serum, with a distinguishably low cycle threshold value standard deviation. The fold-change in relative expression was calculated using the 2^-ΔΔ^Ct method [37].

### Statistical analysis

Statistical analysis was performed using SAS software, version 9.4 (SAS Institute Inc., Cary, North Carolina, USA). Continuous data are expressed as means ± standard error of the mean (SEM) for normally distributed variables or median and interquartile range (IQR) for non-normally distributed variables. Differences in animal characteristics and the vascular reactivity dose-response curves were determined by repeated-measures analysis of variance (ANOVA) followed by Tukey *post-hoc* tests. The sensitivity (EC_50_) and the maximal (E_max_) responses were determined from regression analysis of logistic sigmoid function curves (GraphPad Software Inc, San Diego, CA). A two-way ANOVA followed by a Tukey *post-hoc* test was used to determine differences in inflammatory markers, endothelial activation markers and the EC_50_ and E_max_ responses, where the main effects were group and sex. A Kruskal-Wallis test was used to determine differences in miRNA expressions between groups. Except for body weight, there were no sex differences in inflammatory cytokine concentrations and vascular measurements, hence the male and female data were pooled to increase statistical power. To determine whether miRNAs are associated with circulating inflammatory markers, endothelial activation markers and vessel relaxation responses, bivariate and multivariate linear regression analyses were performed. As body mass and blood pressure are known confounders to vessel function, these variables were included in multivariate regression analyses. Non-normally distributed variables were log-transformed prior to inclusion in regression analysis. A p-value < 0.05 was considered statistically significant.

## Results

### Animal characteristics

The characterization of the animal model has been reported [30]. Briefly, for the animals included in this study the onset of arthritis, as indicated by joint swelling and oedema, occurred in the CIA and CIA+etanercept groups within 21–28 days after the primary immunization [30]. At termination arthritis scores remained increased compared to the control group in both the CIA and CIA+etanercept groups, however, paw thickness at the tarsometatarsal and ankle joints were similar between the CIA+etanercept and control groups [30]. The body weight, and systolic and diastolic blood pressures were similar between the groups at baseline and termination (all p>0.05; Table 1). The serum concentrations of TNF-α, IL-6 and CRP were increased in the CIA and CIA+etanercept groups compared to the control group (all p < 0.05; Table 1). Compared to the CIA group, the serum concentrations of CRP were lower (p = 0.03; Table 1) and TNF-α tended to be lower (p = 0.07) in the CIA+etanercept group.

**Table 1:** Body weights, food intake and tail-cuff blood pressure at baseline and termination.

	Control (n = 24)	CIA (n = 24)	CIA+etanercept (n = 16)
**Baseline**			
Body weight (g)	431 ± 25	387 ± 25	371 ± 33
Systolic blood pressure (mm Hg)	128 ± 2	128 ± 2	129 ± 2
Diastolic blood pressure (mm Hg)	89 ± 1	89 ± 1	89 ± 2
**Termination**			
Body weight (g)	494 ± 25	419 ± 24	388 ± 33
Systolic blood pressure (mm Hg)	127 ± 2	129 ± 2	130 ± 2
Diastolic blood pressure (mm Hg)	86 ± 2	89 ± 1	86 ± 2
*Circulating inflammatory markers*			
TNF-α (pg/ml)	98.7 ± 9.9	177.9 ± 8.7*	145.6 ± 11.6*
IL-6 (pg/ml)	18.4 ± 2.1	31.5 ± 1.9*	27.8 ± 2.5*
CRP (ng/ml)	0.12 ± 0.04	0.60 ± 0.04*	0.43 ± 0.05*^†^

Data presented as mean ± SEM.

*p<0.05 versus control.

^†^*p*<0.05 versus CIA. CIA; collagen-induced arthritis; TNF-α: Tumor necrosis factor alpha; IL-6: Interleukin 6; CRP: C reactive protein.

### Biomarkers of endothelial function

**Fig 1:**
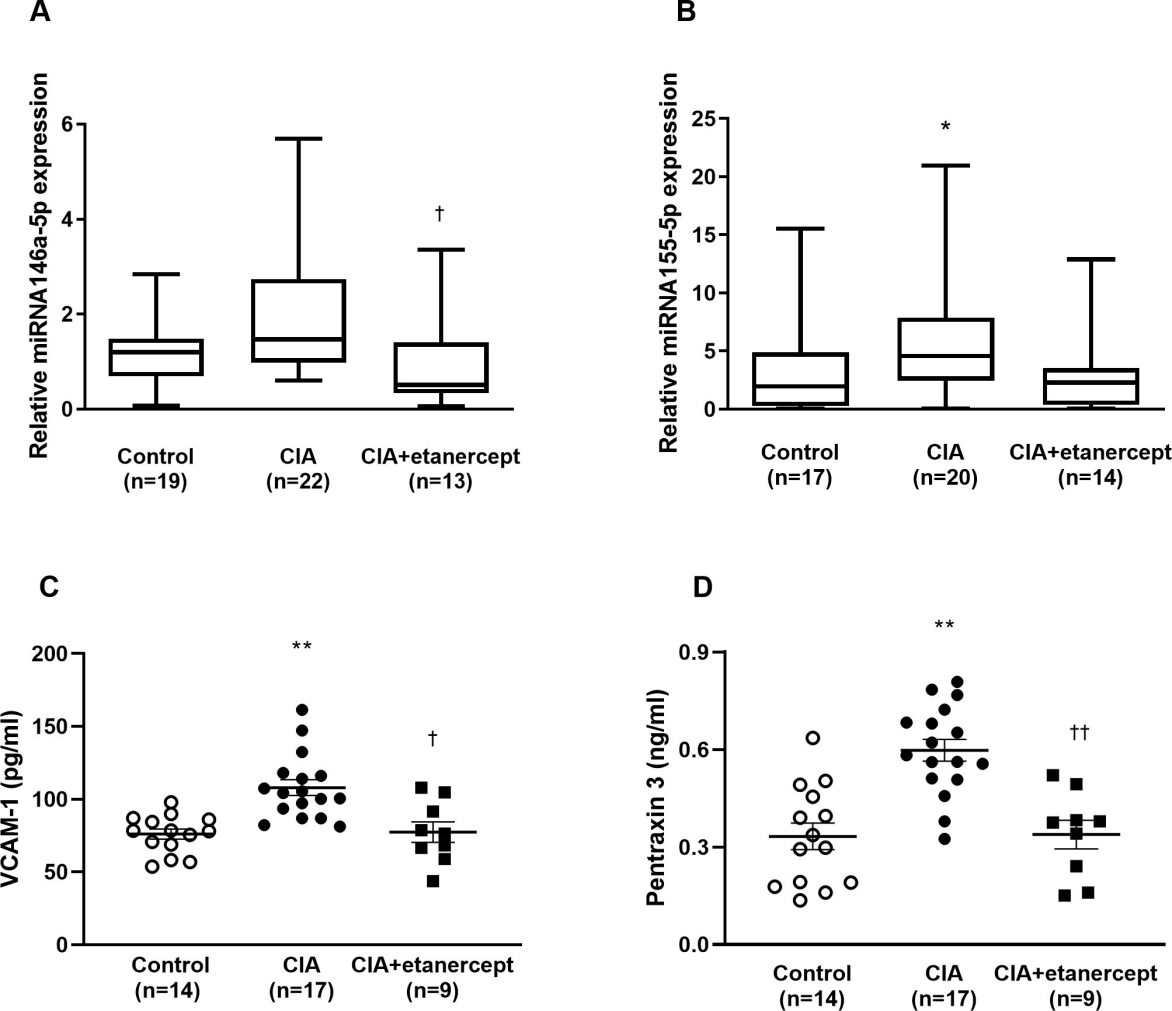
Circulating concentrations of biomarkers of endothelial function in control, CIA and CIA+etanercept groups. Circulating concentrations of miR146a-5p (A), miR 155-5p (B), VCAM-1 (C) and PTX-3 (D) in control, CIA, and CIA+etanercept groups. Data presented as mean ± SEM or median (IQR). *p<0.05 versus control, **p<0.001 versus control. ^†^p<0.05 versus CIA, ^††^p<0.001 versus CIA, using a Kruskal-Wallis test or a two-way ANOVA followed by Tukey *post-hoc* tests. CIA: collagen-induced arthritis; VCAM-1: Vascular adhesion molecule-1; PTX-3: Pentraxin-3.

The relative expression of miRNA-146a-5p was significantly higher in the CIA group compared to the CIA+etanercept group (p = 0.006, Fig 1A). The relative expression of miRNA-155-5p was significantly higher in the CIA group compared to the control group (p = 0.048, Fig 1B). The expression of miRNA-155-5p in the CIA group was higher than the CIA+etanercept group but did not reach statistical significance (p = 0.09, Fig 1B).

Circulating VCAM-1 concentrations were significantly higher in the CIA group compared to the control and the CIA+etanercept groups (p = 0.0003 and p = 0.003 respectively, Fig 1C). Circulating PTX-3 concentrations were significantly higher in the CIA group compared to the control and the CIA+etanercept groups (p = 0.001 and p = 0.005 respectively, Fig 1D). To determine whether inflammation was responsible for the increase in these endothelial function markers, we determined the association between CRP concentrations and VCAM-1 and PTX-3 concentrations. Circulating CRP concentrations were directly associated with VCAM-1 concentrations (r (95%CI) = 0.29 (-0.01–0.55); p = 0.048) and PTX-3 concentrations (r (95%CI) = 0.33 (0.03–0.58); p = 0.03).

### Vascular reactivity

**Fig 2:**
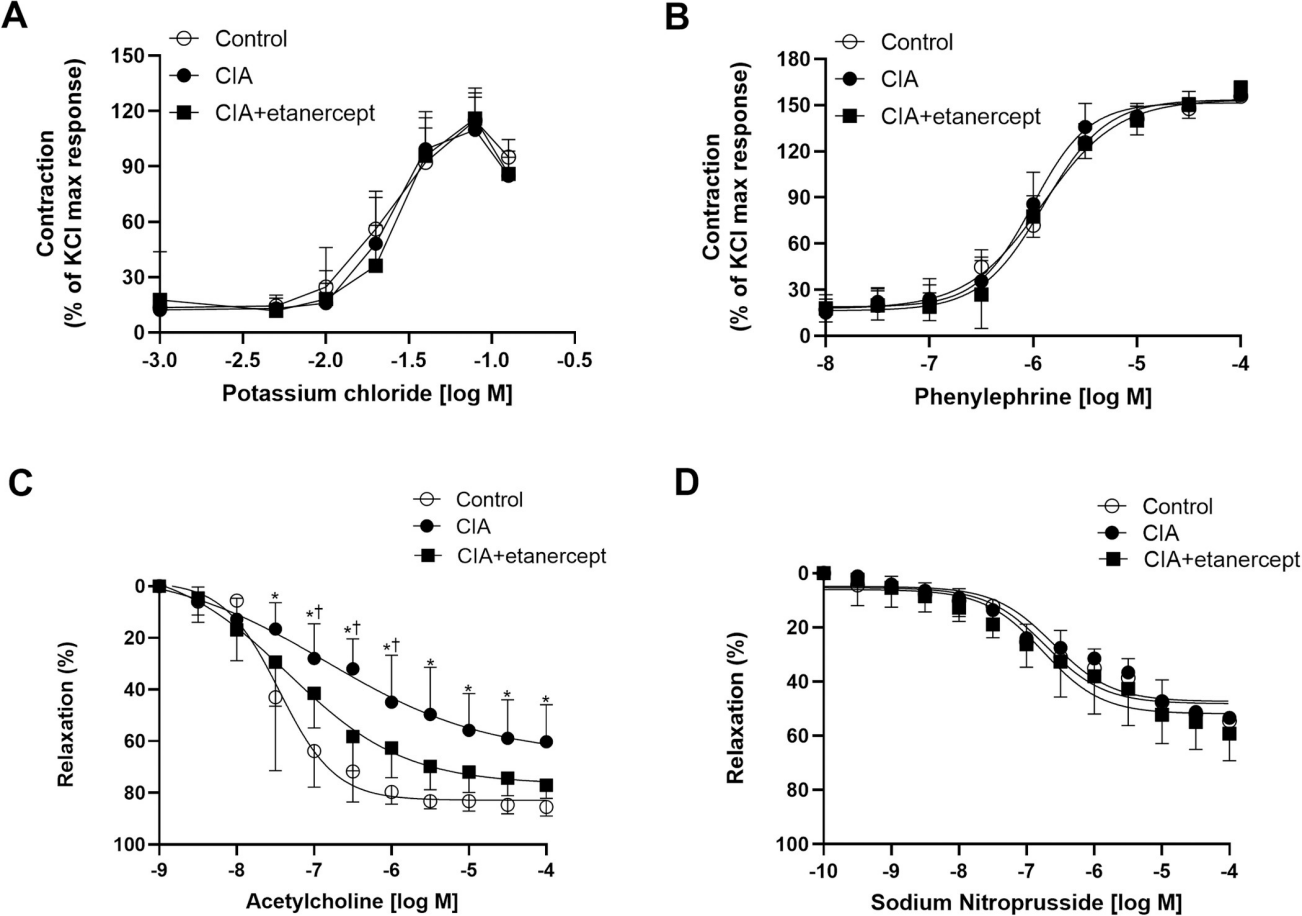
The effects of inflammation and TNF-α inhibitor treatment on vessel responses to vasoactive substances. The cumulative dose-response curves to (**A**) potassium chloride, (**B**) phenylephrine **(C)** acetylcholine and **(D)** sodium nitroprusside in the second branch of mesenteric arteries, in control, CIA and CIA+etanercept groups. Contractile responses are expressed as a percentage of the KCl maximal response. Relaxation responses are expressed as a percentage of the Phe (10 μM)-induced contraction. Data expressed as means ± SEM. *p<0.01 control and CIA+etanercept versus CIA, ^†^p<0.05 CIA+etanercept versus control; using repeated-measures ANOVA followed by Tukey *post-hoc* tests.

The reference contraction induced by potassium chloride (KCl; 80mM) was similar between the experimental groups (all p>0.05). There were no differences in the KCl-induced and Phe-induced contractile responses between the groups (p>0.05; Fig 2A and 2B). Phe (10 μM)-induced contractions were similar between the groups before the addition of acetylcholine (ACh) (all p>0.05) or sodium nitroprusside (SNP) (all p>0.05).

**Fig 3:**
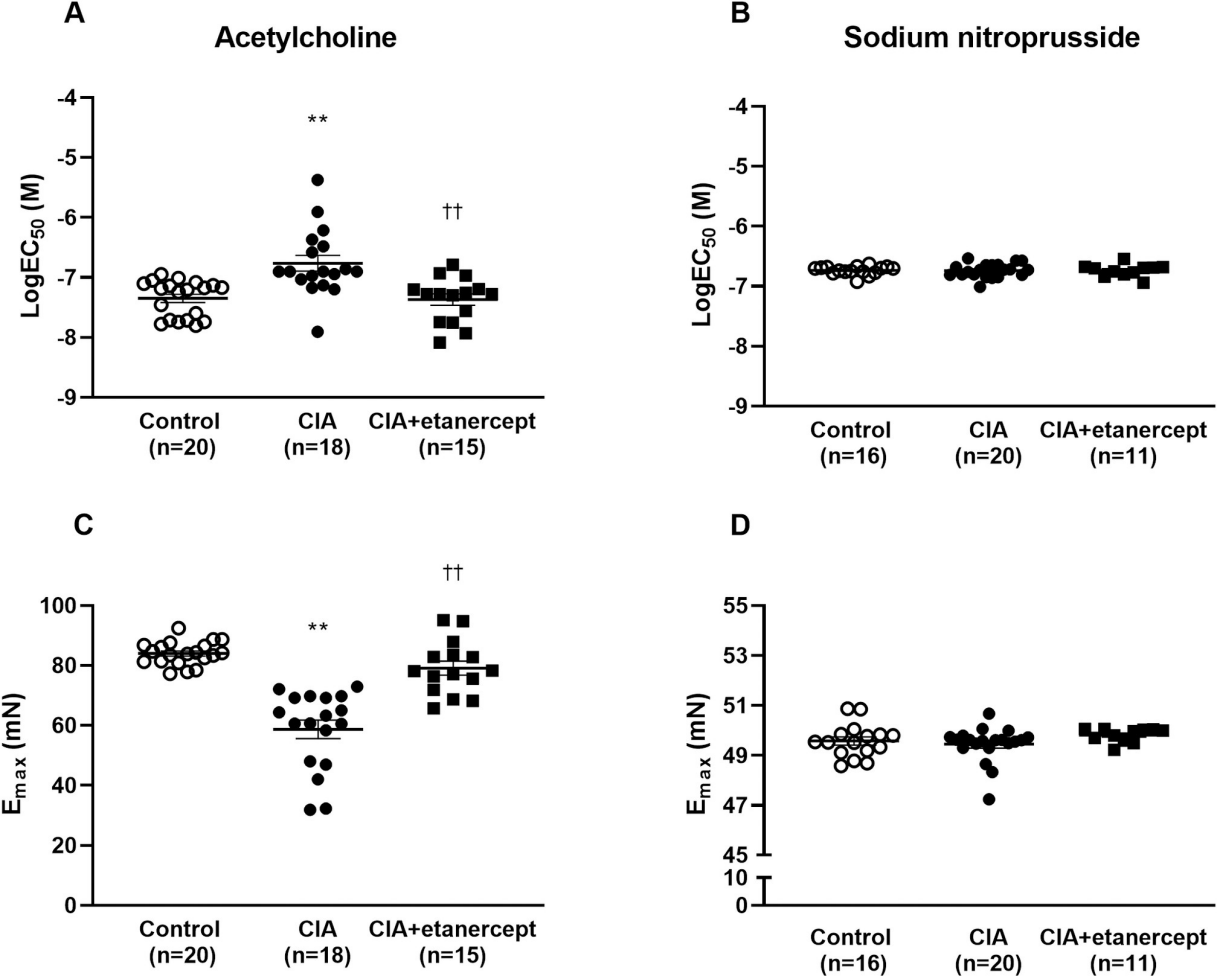
The effects of TNF-α inhibitor treatment on the sensitivity (LogEC_50_) and maximum responses (E_max_) to vasodilatory substances in mesenteric arteries. The sensitivity (LogEC_50_) to acetylcholine (A) and sodium nitroprusside (B) and the maximal relaxation response (E_max_) to acetylcholine (C) and sodium nitroprusside (D) in the second branch of mesenteric arteries, in control, CIA and CIA+etanercept groups. Data expressed as means ± SEM. **p<0.001 versus control; ^††^ p<0.001 versus CIA; using two-way ANOVA and Tukey *post-hoc* tests.

The ACh-induced relaxation responses at 10^−7.5^ to 10^-4^M were significantly impaired in the CIA group compared to the control group and the CIA+etanercept group (all p<0.01; Fig 2C). The ACh-induced relaxation responses at 10^−7^ to 10^-6^M were significantly impaired in the CIA+etanercept group compared to the control group (all p<0.05; Fig 2C). There were no differences in the relaxation responses (Fig 2D), logEC_50_ and E_max_ (Fig 3) for SNP-induced relaxation between the groups in mesenteric arteries (all p>0.05).

The logEC_50_ was significantly lower in the CIA group compared to the control and CIA+etanercept groups (p = 0.0008 and p = 0.001, respectively; Fig 3). The E_max_ of ACh-induced relaxation was significantly reduced in the CIA group compared to the control and CIA+etanercept groups (both p<0.0001; Fig 3).

To determine whether VCAM-1 and PTX-3 were indeed markers of endothelial function, we determined the associations between these endothelial function markers and vascular reactivity measures. VCAM-1 concentrations were directly associated with mesenteric artery ACh-induced sensitivity (logEC_50_) (r (95%CI) = 0.48 (0.12–0.72); p = 0.01) and with mesenteric artery ACh-induced maximum relaxation responses (E_max_) (r (95%CI) = -0.52 (-0.75- -0.18); p = 0.003). When adjusting for body mass, blood pressure and CRP, VCAM-1 concentration remained associated with mesenteric artery ACh-induced sensitivity (logEC_50_) (r (95%CI) = 0.43 (0.02–0.71); p = 0.04) and with mesenteric artery ACh-induced maximum relaxation responses (r (95%CI) = -0.61 (-0.81- -0.26); p = 0.001). Similarly, PTX-3 concentrations were directly associated with mesenteric artery ACh-induced sensitivity (logEC_50_) (r (95%CI) = 0.65 (0.37–0.83); p<0.001) and with ACh-induced maximum relaxation responses (E_max_) (r (95%CI) = -0.41 (-0.68- -0.04); p = 0.03). These associations were materially unaltered when adjusting for body mass, blood pressure, and CRP (logEC_50_: r = 0.62 (0.27–0.81); p = 0.001 and E_max_: r = -0.49 (-0.75- - 0.10); p = 0.01).

### Association between microRNAs and measures of circulating inflammation, endothelial function, and vascular relaxation

In unadjusted regression analysis, the relative expression of miRNA-146a-5p was associated with increased circulating CRP and TNF-α concentrations (both p = 0.01; Table 2). When adjusting for body mass and blood pressure, the association between miRNA-146a-5p and circulating CRP and TNF-α concentrations remained significant (p = 0.009 and p = 0.01, respectively; Table 2). Increased miRNA-146a-5p expression was associated with increased circulating PTX-3 concentrations (p = 0.03), but not with circulating VCAM-1 concentrations (p = 0.26; Table 2). When adjusting for body mass and blood pressure, miRNA-146a-5p remained significantly associated with PTX-3 (std β (SE) = 0.48 (0.16); p = 0.01). When additionally adjusting for CRP concentrations, these associations remained materially unaltered (std β (SE) = 0.41(0.17); p = 0.02). In unadjusted regression analysis, the relative expression of miRNA-146a-5p was positively associated with mesenteric artery ACh-induced sensitivity (logEC_50_) (p = 0.04) and this association remained significant in multivariate regression analyses (p = 0.04; Table 2).

**Table 2:** Associations between relative microRNA expressions and markers of inflammation, endothelial function and vascular relaxation.

	miRNA-146a-5p*			miRNA-155-5p*		
	N	std β (SE)	p	n	std β (SE)	p
Unadjusted						
*Circulating inflammatory markers*						
CRP	57	0.34(0.13)	**0.01**	54	0.28(0.13)	**0.03**
TNF- α	54	0.31(0.12)	**0.01**	52	0.14(0.13)	0.26
IL-6	55	0.23(0.13)	0.09	52	0.04(0.13)	0.77
*Circulating endothelial function markers*						
VCAM-1	35	0.17(0.15)	0.26	31	0.03(0.13))	0.81
PTX-3	35	0.35(0.15)	**0.03**	31	0.03(0.17)	0.87
*Vascular relaxation markers*						
Mesenteric artery ACh EC_50_	41	0.35(0.17)	**0.04**	41	0.16(0.17)	0.35
Mesenteric artery ACh E_max_	41	-0.23(0.18)	0.20	41	-0.43(0.16)	**0.009**
Multivariate adjusted ^#^						
*Circulating inflammatory markers*						
CRP	57	0.36(0.13)	**0.009**	54	0.39(0.12)	**0.003**
TNF- α	54	0.33(0.13)	**0.01**	52	0.29(0.13)	**0.03**
IL-6	55	0.25(0.14)	0.08	52	0.16(0.13)	0.19
*Circulating endothelial function markers*						
VCAM-1	35	0.16(0.17)	0.34	31	0.11(0.15)	0.47
PTX-3	35	0.48(0.16)	**0.007**	31	0.16(0.17)	0.36
*Vascular relaxation markers*						
Mesenteric artery ACh EC_50_	41	0.38(0.18)	**0.04**	41	0.18(0.17)	0.28
Mesenteric artery ACh E_max_	41	-0.25(0.19)	0.20	41	-0.43(0.16)	**0.009**

* Logarithmically transformed variables.

^#^ adjusted for body mass and blood pressure. CRP: C reactive protein; TNF-α: Tumour necrosis factor alpha; IL-6: Interleukin-6; VCAM-1: Vascular adhesion molecule-1; PTX-3: Pentraxin-3; ACh: Acetylcholine.

The relative expression of miRNA-155-5p was associated with increased circulating CRP concentrations in univariate (p = 0.03) and in multivariate regression models (p = 0.003; Table 2). No significant associations were observed between the relative expression of miRNA-155-5p and circulating VCAM-1 or PTX-3 concentrations (both p>0.05). The relative expression of miRNA-155-5p was negatively associated with mesenteric artery ACh-induced maximum relaxation responses in unadjusted analysis (p = 0.009) and when adjusting for body mass and blood pressure (p = 0.0009; Table 2). When further including CRP concentrations in the regression model, the association between miRNA-155-5p and maximal mesenteric artery relaxation was no longer significant (std β (SE) = -0.32 (0.17); p = 0.07).

## Discussion

This study investigated whether inhibiting circulating TNF-α impact the relative expression of miRNAs in rats exposed to inflammation, and whether relative miRNA expressions are related to endothelial function and vascular relaxation responses. The main findings of the present study are that TNF-α inhibition ameliorated inflammation-induced aberrant expression of miRNA-146a-5p and miRNA-155-5p. In turn, TNF-α inhibition reduced inflammation-induced increases in serum concentrations of VCAM-1 and PTX-3 and impairments in endothelium dependent vascular responses in mesenteric arteries. MiRNA-146a-5p was independently associated with PTX-3 expression, a marker of vascular inflammation. This suggests that miRNA-146a-5p may be involved in the regulation of vascular inflammation that is mediated by TNF-α. Increased miRNA-155-5p expression was associated with impaired ACh-induced maximum relaxation responses in mesenteric arteries. These results confirm that NO-dependent vasodilation may at least, in part, be mediated by inflammation-induced upregulation of miRNA-155-5p. Taken together our results suggest that inflammation-induced increased expression of miRNA-146a-5p and miRNA-155-5p may be mediated by TNF-α and that these miRNAs are differentially associated with distinct processes involved in inflammation-induced impairments in vessel function.

In the present study, circulating miRNA-146a-5p expression was associated with higher serum TNF-α and CRP concentrations. MiRNA-146a-5p is produced by hematopoietic stem and progenitor cells, myeloid cells and various leukocytes [38], and its expression is upregulated in response to systemic inflammation [39]. Inflammatory cytokines, including TNF-α, induce miR-146a-5p expression via activation of the NF-kB signalling pathway [19]. In turn, several studies suggest that miR-146a-5p is involved in negative feedback control of TNF-α induced inflammation by targeting specific NF-kB intermediary molecules [19, 40]. However, controversy exists in the literature, with some studies reporting upregulated [41, 42], while others report downregulated miR-146a-5p expression [43, 44] in inflammatory conditions.

In RA patients, several studies reported increased miRNA-146a-5p expression [45, 46]. Although there is evidence of strong relations between increased circulating TNF-α concentrations and miRNA-146a-5p expression in RA, the effects of TNF-α inhibitor treatment on miRNA-146a-5p expression is controversial [43, 45–47]. In the current study, when blocking circulating TNF-α, we showed a reduced expression of miRNA-146a-5p, despite a high circulating TNF-α concentration. Nevertheless, TNF-α inhibitor treatment is aimed specifically at inhibiting the binding of circulating TNF-α to its receptor and receptor inhibition is commonly accompanied by increased circulating concentrations of the cognate ligand [48]. Our findings therefore confirm previous reports that inflammation-induced upregulation of miRNA-16a-5p may be mediated by TNF-α.

Similar to previous reports, in the present study TNF-α inhibition prevented inflammation induced increases in VCAM-1 and PTX-3 concentrations, suggesting decreased endothelial activation and inflammation following TNF-α inhibition [49]. In the present study, miRNA-146a-5p was not associated with VCAM-1. This is in line with previous reports that miRNA-146a-5p may not be a risk marker of atherosclerosis [50, 51]. However, expression of circulating miRNA-146a-5p was related to circulating PTX-3 concentrations, indicating that in an inflammatory model, miRNA-146a-5p may be related to vascular inflammation [24]. Importantly, although the association between circulating PTX-3 concentrations and miRNA-146a-5p expression remained significant following adjustment for circulating CRP concentrations, the strength of the association was reduced. This suggests that inflammation may indeed be a strong driver of the relationship between miRNA-146a-5p and endothelial inflammation. Nevertheless, miRNA-146a-5p may be a biomarker of endothelial dysfunction independent of systemic inflammation. Hence miRNA-146a-5p may hold potential as a therapeutic target in vascular inflammation in RA.

In the current study, inflammation reduced the sensitivity of the mesenteric artery to vasoactive substances, that were offset by blocking circulating TNF-α. These results are in accordance with previous reports that TNF-α induces alterations in the receptor expression of various vasoactive substances [52, 53]. Moreover, we showed that miRNA-146a-5p expression was associated with the sensitivity of mesenteric arteries (LogEC_50_) to acetylcholine. Hence, our results indicate that TNF-α inhibition downregulate general vascular inflammatory responses that may contribute to endothelial dysfunction, possibly via the regulation of miRNA-146a-5p. Furthermore, blood pressure was not altered by inflammation or TNF-α inhibitor therapy, and the associations between miRNA-146a-5p and PTX-3 and mesenteric artery sensitivity were independent of blood pressure. Taken together, these results suggest that TNF-α inhibition may improve endothelial function and miRNA146a-5p may be involved in the regulation of endothelial inflammation via mechanisms independent of blood pressure.

In the present study, similar to reports in RA patients [23, 54], the relative expression of miRNA-155-5p was significantly upregulated following exposure to high-grade inflammation and was related to higher serum CRP concentrations. Indeed, inflammatory cytokines are the primary stimulus for increased expression of miRNA-155-5p [18, 38]. In turn, miRNA-155-5p expression drives the inflammatory response of immune cells in a positive feedback manner, by repressing the production of inhibitory proteins that act on the NF-kB pathway [55]. However, blocking circulating TNF-α in the present study, prevented inflammation-induced upregulation of miRNA-155-5p. Similarly, blocking circulating TNF-α prevented the inflammation-induced impairment in ACh-induced relaxation. In this regard, it is well known that TNF-α decreases the expression of the endothelial nitric oxide (eNOS) enzyme [56], which results in decreased NO production and ultimately impaired vasodilation [56]. Similar to our findings, previous studies have shown improved ACh-induced relaxation responses following TNF-α inhibitor therapy [29, 57]. Therefore, our results confirm that inflammation-induced, endothelial dependent impaired vessel relaxation may be mediated via TNF-α. Furthermore, previous reports suggest that miRNA-155-5p may be involved in the regulation of inflammation induced impairments in endothelial dependent, but not endothelial independent vasorelaxation [23]. Indeed, in the present study we showed that circulating miRNA-155-5p expression was associated with impaired Ach-induced maximum relaxation responses, but not SNP induced relaxation responses. Moreover, the association between miRNA-155-5p and endothelial dependent vessel relaxation was independent of blood pressure. However, when adjusting for circulating CRP, the association between miR-155-5p expression and mesenteric artery maximal relaxation was no longer significant. Therefore, these results suggest that inflammation is a key driver of upregulated miR-155-5p expression, but that in inflammatory conditions miRNA-155-5p may be involved in the regulation of vasorelaxation via mechanisms that are independent of blood pressure.

This study has limitations. We used a recombinant humanized TNF-α dimer. Although previous rat studies using similar TNF-α inhibitors have shown significant effects on inflammation-induced outcomes [58], future studies should consider using rat specific TNF-α inhibitor therapy. We determined the expression of two circulating miRNAs relative to surrogate measures of endothelial function. Inflammatory cytokines may regulate endothelial function through upregulated miRNA expression from circulating leukocytes and hematopoietic cells, and therefore we measured the expression of miRNAs in serum. Nevertheless, miRNAs are ubiquitously expressed and hence measuring circulating miRNAs levels may reflect a compensatory upregulation in response to inflammation, rather than endothelial miRNA expression. However, *in vitro* studies using human umbilical vein endothelial cell (HUVEC) lines have reported similar upregulated expression of miR-155-5p and miR-146a-5p following exposure to TNF-α [59, 60] and strong relations between circulating and tissue expressions of miRNAs have previously been reported [23, 24, 61]. Similarly, circulating PTX-3 concentrations may have originated from various cell types. Nevertheless, endothelial PTX-3 is highly expressed in inflammatory conditions [62–64]. Although we have shown independent associations between miRNAs and markers of endothelial function, correlations do not infer causation and these results may show reciprocal relations. Moreover, the interpretations of our results may have been strengthened by measuring downstream intracellular adaptor proteins and NO-dependent signalling mechanisms to explore the mechanism whereby TNF-α inhibition impacted markers of endothelial dysfunction independent of changes in blood pressure.

In conclusion, TNF-α inhibition prevented inflammation-induced upregulation of miRNA-146a-5p, miRNA-155-5p, PTX-3 and VCAM-1, and improved vasorelaxation. MiRNA-146a-5p expression was associated with vessel inflammation, independent of circulating inflammation, while miRNA-155-5p was associated with mesenteric artery vasorelaxation. MiRNAs may be relevant biomarkers of vessel dysfunction in inflammatory conditions. Future studies should investigate the potential of miRNAs as therapeutic targets for protecting against endothelial dysfunction in inflammatory conditions.
